# Engaging community pharmacists in the primary prevention of cardiovascular disease: protocol for the Pharmacist Assessment of Adherence, Risk and Treatment in Cardiovascular Disease (PAART CVD) pilot study

**DOI:** 10.1186/1472-6963-10-264

**Published:** 2010-09-07

**Authors:** Kevin P Mc Namara, Johnson George, Sharleen L O'Reilly, Shane L Jackson, Gregory M Peterson, Helen Howarth, Michael J Bailey, Gregory Duncan, Peta Trinder, Elizabeth Morabito, Jill Finch, Stephen Bunker, Edward Janus, Jon Emery, James A Dunbar

**Affiliations:** 1 Centre for Medicine Use and Safety, Department of Pharmacy Practice, Faculty of Pharmacy and Pharmaceutical Sciences, Monash University, 381 Royal Parade, Parkville, VIC 3052, Australia; 2 Greater Green Triangle University Department of Rural Health, Flinders and Deakin Universities, Deakin University, Princes Hwy, Warrnambool, VIC 3280, Australia; 3 Centre for Physical Activity and Nutrition Research, Faculty of Health Medicine Nursing & Behavioural Sciences, Deakin University, 221 Burwood Highway, Burwood, Victoria 3125, Australia; 4 Unit for Medication Outcomes Research and Education, School of Pharmacy, University of Tasmania, Churchill Avenue, Sandy Bay, TAS 7005, Australia; 5 Department of Epidemiology and Preventive Medicine, Monash University, Alfred Hospital, Commercial Road, VIC 3004, Australia; 6 Peter James Centre, Eastern Health Clinical School, Faculty of Medicine, Nursing and Health Sciences, Monash University, Mahoney's Road, Burwood East, Victoria 3151 Australia; 7 Department of Medicine, University of Melbourne, Western Hospital, Gordon Street, Footscray VIC 3011; 8 School of Primary, Aboriginal and Rural Health Care. The University of Western Australia (M501), 35 Stirling Highway, Crawley, Western Australia 6009, Australia

## Abstract

**Background:**

Cardiovascular disease (CVD) is the leading cause of death globally. Community pharmacist intervention studies have demonstrated clinical effectiveness for improving several leading individual CVD risk factors. Primary prevention strategies increasingly emphasise the need for consideration of overall cardiovascular risk and concurrent management of multiple risk factors. It is therefore important to demonstrate the feasibility of multiple risk factor management by community pharmacists to ensure continued currency of their role.

**Methods/Design:**

This study will be a longitudinal pre- and post-test pilot study with a single cohort of up to 100 patients in ten pharmacies. Patients aged 50-74 years with no history of heart disease or diabetes, and taking antihypertensive or lipid-lowering medicines, will be approached for participation. Assessment of cardiovascular risk, medicines use and health behaviours will be undertaken by a research assistant at baseline and following the intervention (6 months). Validated interview scales will be used where available. Baseline data will be used by accredited medicines management pharmacists to generate a report for the treating community pharmacist. This report will highlight individual patients' overall CVD risk and individual risk factors, as well as identifying modifiable health behaviours for risk improvement and suggesting treatment and behavioural goals. The treating community pharmacist will use this information to finalise and implement a treatment plan in conjunction with the patient and their doctor. Community pharmacists will facilitate patient improvements in lifestyle, medicines adherence, and medicines management over the course of five counselling sessions with monthly intervals. The primary outcome will be the change to average overall cardiovascular risk, assessed using the Framingham risk equation.

**Discussion:**

This study will assess the feasibility of implementing holistic primary CVD prevention programs into community pharmacy, one of the most accessible health services in most developed countries.

**Trial registration:**

Australia and New Zealand Clinical Trial Registry Number: ACTRN12609000677202

## Background

Cardiovascular disease (CVD) is the leading cause of death globally, accounting for an estimated 17.1 million deaths per annum - 29% of all deaths [1]. The relative contribution of CVD to the burden of disease remains high in countries with low-, middle-, and high incomes [1]. Despite significant gaps in research, there is sufficient evidence (particularly from developed nations such as the United States and United Kingdom) to suggest that the primary prevention of CVD represents a cost effective approach to reducing this burden [2].

There is a cluster of highly prevalent CVD risk factors in many developing and most developed countries including Australia which can be controlled or avoided: tobacco smoking, hypertension, overweight and obesity, physical inactivity, dyslipidaemia (high cholesterol), poor nutrition and diabetes [1,3]. Significant evidence-treatment gaps remain in the management of each of these, culminating in substantially elevated incidences of CVD in most countries. Community pharmacists are highly accessible health professionals in most communities and have demonstrated an effective role in the management of several of these risk factors [4-7]. Guidelines on primary CVD prevention now highlight the importance of overall CVD risk assessment rather than traditional individual risk factor assessment, resulting in a much greater emphasis on multiple risk factor management [8-13].

The expertise of community pharmacists in medicines management including adherence screening and management, and a growing role in health promotion has resulted in several studies demonstrating effective pharmacist involvement with multiple CVD risk factor management [14-16]. However, we are not aware of any multiple risk factor study specifically examining structured, pharmacist-led disease state management programs for primary CVD prevention in a non-diabetic population. The *Pharmacist Assessment of Adherence, Risk and Treatment in Cardiovascular Disease (PAART CVD) *was developed as a pilot project to address this evidence gap.

This paper describes the research protocol developed for the PAART CVD project, a community-pharmacy based CVD primary prevention pilot project in a non-diabetic, adult population. We also identify the theoretical framework, local issues affecting implementation decisions, and gaps in current knowledge experienced to facilitate future adaptation.

### Goals of research

1. To assess the feasibility of implementing a primary CVD prevention program in a community pharmacy setting

2. To measure changes to cardiovascular health for patients receiving the PAART CVD intervention.

## Methods and Design

### Study design and setting

This is a longitudinal pre- and post-test study to be undertaken with a single cohort of patients. Ten pharmacies from two States (Victoria and Tasmania) will be recruited - five each from rural and metropolitan locations. Each pharmacy will be asked to approach a convenience sample of patients meeting the inclusion criteria, with a goal of recruiting 10 participants per pharmacy. Local general practices and general practice organisations will be informed about the project.

### Outcomes examined

The primary outcome for the intervention will be the average change to estimated 5-year risk of primary onset of CVD between baseline and six months calculated using the New Zealand CVD risk scores [11], a modified version of the Framingham-based risk equation [17]. Secondary health outcomes include change to blood pressure, lipid profile, random blood glucose, waist, body mass index (BMI), and depression score.

### Pharmacist training

Over two consecutive days, pharmacists from recruited pharmacies will attend training focussed on the following key topics:

• health promotion and behavioural change

• overall CVD risk and CVD risk assessment

• medicines adherence

• medicines management for cardiovascular risk factors

• lifestyle modification (diet, physical activity, weight management, alcohol consumption)

• antiplatelet use for primary CVD prevention

• smoking cessation

• managing patients who screen positively for diabetes, alcohol abuse or depression; and

• General Practitioner (GP) and other health professional engagement.

All training, including training materials, will be consistent with relevant national guidelines and will be tailored to suit a generalist community pharmacist audience. Emphasis will be placed on the need for pharmacists to act within their professional competencies and take appropriate action if situations arise outside their competencies (e.g. patient screens positive for depression). Written guidelines will be provided to identify patients with complex or urgent clinical needs that warrant referral to a GP or another health professional (e.g. dietitian). Pharmacists will also be advised on options for referral (e.g. how to access specialist dietary or smoking cessation advice) when they might deem it appropriate.

### Subject eligibility criteria

Inclusion criteria

To be eligible for participation in the study, patients need to be

• aged 50-74 years

• currently taking one or more medicines for cholesterol and/or hypertension, and

• free from established cardiovascular disease, diabetes or a cardiovascular event.

Individuals will be excluded from the study in the following instances:

• patients with a complex debilitating coexisting medical condition

• target organ damage

• any cognitive impairment

• reliance on a carer or living in a residential aged care facility

• recent hospital inpatients who were medical admissions and discharged less than four weeks prior to recruitment

• non-English speaking

• living more than 40 km from a participating pharmacy

• patients who have received a Home Medicines Review (HMR) in the past 12 months

• deemed inappropriate for the intervention by the patient's GP; or

• patients of GPs who express a desire not to have any involvement with this project.

### Patient recruitment

Pharmacists will initially be asked to conduct a simple pre-screening of potential participants based on the inclusion criteria outlined above. The information required (CVD history, use of CVD medicines, and age) is quick and easy to obtain from dispensing records and patient interview. Potential participants expressing interest will be provided with written patient information and an Expression Of Interest (EOI) form to be completed and returned to researchers.

Upon receipt of EOIs or direct patient-initiated contact via a free telephone number, a formal assessment of all eligibility requirements will be undertaken by researchers and a provisional baseline assessment date agreed. If eligible, consent must be obtained to contact their GP in writing and to provide the GP with an opportunity to comment on the appropriateness of participation. Written consent for participation and sharing of health information with relevant health professionals is to be provided by patients prior to baseline assessment.

### Patient data collection

Baseline and final (6 month) patient information will be collected by trained research assistants (RAs). This process will take 60-90 minutes on both occasions and can take place in the patient's home (preferred), or in a private area at the pharmacy or their workplace. Assessment involves anthropometric and Point Of Care (POC) biomedical testing, and an administered questionnaire using validated scales where possible. Lipid profiles and fasting blood glucose levels will be obtained using Cholestech LDX^® ^Analyzers [18,19], in accordance with instructions provided by the manufacturer. This device uses finger-prick testing and provides results within 10 minutes. Quality control samples will be used to validate the machine prior to measurements. Patients will be advised that the most accurate results for cholesterol and blood glucose levels would be obtained by fasting 12-16 hours prior to the initial assessment interview and testing, and to take their medications as normal on the day of clinical assessment. Fasting time will be recorded to aid clinical interpretation.

When the patient is rested, blood pressure (BP) will be measured using an Omron 1A1B^® ^automated BP monitor in accordance with WHO MONICA Monitoring of Trends and Determinants of Cardiovascular Diseases and the European Health Risk Monitoring protocols [20,21] amended for use with an automated monitor. The average of two measurements taken 2-3 minutes apart will be used, but if systolic BP measurements differ by 10 mmHg or greater, or diastolic BP differ by 6 mmHg or greater, a third reading will be taken. In this instance the average of the two closest systolic and two closest diastolic BP measurements will be used to define the BP. The RA will advise patients to seek immediate medical attention if systolic BP is 180 mmHg or greater, or if diastolic BP is 110 mmHg or greater. Weight, height, and waist and hip circumferences will be measured in accordance with the EHRM protocol [21].

The remainder of the assessment will consist of a researcher-administered questionnaire (all interviewing researchers have pharmacy qualifications). This questionnaire, which includes several validated survey instruments, covers a range of health indicators:

• demographic and social information, including some questions based on the WHO MONICA (MONItoring of CArdiovascular events) protocol [21], as adapted for the Greater Green Triangle Chronic Disease Risk Factor Studies [22]

• brief patient medical history and family history of CVD events

• details of current medicines

• details of recent testing for CVD risk factors prior to baseline

• adherence to current medications advice using the Tool for Adherence Behaviour Screening (TABS) [23] and the Morisky scale [24]

• smoking, physical activity and weight management status - including some screening questions from the 'Lifescripts' program [25] and questions from surveys used by the Greater Green Triangle Chronic Disease Risk Factor Studies [22]

• nutrition and adherence to national dietary guidelines, based on Dietary Guidelines for Australian Adults (2003) [26] and using a number of questions from the National Nutrition Survey 1995 [27], and the Cancer Council of Victoria Food Frequency Questionnaire [28]

• screening for excess alcohol consumption using the AUDIT C tool [29]

• screening for depression using the Center for Epidemiological Study of Depression Scale (CES-D) [30]

• quality of life measure using the SF-12v2 Health Survey™ (Ware et al, 2007) [31].

Pharmacists will be asked to supply medicines dispensing data for the six months' period prior to baseline data collection and the six months prior to follow up data collection. To account for medicines collected elsewhere, patients will be asked during their interviews to recall any quantities of medicines obtained elsewhere for the specified period.

### Intervention part one: accredited pharmacist review

Using relevant national clinical guidelines as the benchmark for management, pharmacists accredited to undertake collaborative, structured medicines management reviews in community settings (known as 'Home Medicines Reviews') will produce a written report for each patient highlighting the estimated 5-year CVD risk, suboptimal CVD risk factors, and medication adherence and other medicines use issues (see additional file 1 for template). The report will also suggest patient treatment goals and opportunities for beneficial lifestyle changes and improved medicines management to achieve goals. This report will be provided to the patient's community pharmacist with summaries for the patient and their general practitioner.

### Intervention part two: community pharmacist facilitating patient change

Community pharmacists will be trained to deliver their interventions in accordance with the Health Action Process Approach (HAPA) [32] to behaviour change over five counselling sessions conducted at monthly intervals. The emphasis in counselling progresses from change motivation initially (via improved self-efficacy, belief in the need for change and belief that change will generate positive outcomes), through to change maintenance and relapse prevention strategies. Written, achievable goals will be encouraged.

The first session with the patient will prioritise basic health education regarding individual CVD risk and the benefits of potential treatments. It also establishes acceptable goals for the treatment process through patient consultation, and how these might be achieved. Finally, the pharmacist will discuss with the patient any specific medication changes identified in the baseline report that are recommended to improve adherence to CVD guidelines. Community pharmacists will not be trained or asked to make interventions related specifically to diabetes or mental health issues, but will be alerted to any suboptimal assessment results in these areas and asked to discuss with the patient the potential need for GP input. Such issues will also be identified in the baseline assessment summary provided to the GP.

If a patient's overall 5-year CVD risk score is 5% or less (considered very low risk) they will be advised to discuss with their pharmacist whether they are likely to benefit from continuing with the intervention. The decision to continue will be left to the pharmacist and the patient. Pharmacists will be expected to assess and document patient motivation to undertake various medication and lifestyle changes. Following discussion with each patient, the pharmacist will then forward the clinical summary to the patient's GP with any additional comments considered relevant.

Subsequent sessions will involve: ensuring necessary changes to medicines have been made; monitoring of medicines adherence especially for new medicines; linking patients with local health and other services that provide relevant patient support; initiating lifestyle change and supporting maintenance and relapse prevention. Throughout these sessions, patient progress towards goals will be continually reassessed, as will be the goals themselves. GP input to patient treatment plans will also be invited.

### Primary outcomes

The primary outcome for the PAART CVD intervention is the average change to overall estimated 5-year risk of CVD onset, calculated using the New Zealand adaptation of the Framingham-based CVD risk score [11,17].

### Secondary outcomes

Other outcomes examined include:

• Changes to individual modifiable CVD risk factors (blood pressure, lipid profile, Body Mass Index, waist circumference, smoking status, depression)

• changes in medicines adherence assessed using Morisky [24] and TABS scales [23]; and

• changes in key health behaviours (physical activity, diet, alcohol intake, weight management).

Additional outcomes to be assessed include stakeholder satisfaction (GP, pharmacist, patient) and process audit based on community pharmacist documentation of counselling sessions.

### Sample size

With 80 subjects this study would have a 90% power to show an absolute reduction in risk of 1% based on a standard deviation in the change of risk of 2.7% with a two sided p-value of 0.05. To account for potential subject drop out and loss to follow-up of 20% we will aim to recruit 100 subjects.

### Statistical analysis

Univariate comparisons between groups will be conducted using chi-square test for equal proportion (or Fisher's exact tests where numbers are small) and reported as numbers and percentages. Continuous normally distributed variables will be compared using Student's t-tests and reported as means (standard error) whilst non-parametric data will be compared using Wilcoxon rank-sum tests and reported as medians (interquartile range). Pairwise differences between pre and post values will be calculated using paired t-tests for normally distributed data and Wilcoxon sign rank tests for non-normally distributed data. To account for the large number of comparisons being made, we will employ a reduced 2-sided p-value of 0.01 to indicate clear statistical significance.

If any parameter from POC finger prick testing is found to be outside the measurable range for the machine, the value of the closest measurable limit will be given to that patient.

Health behaviours will largely be measured using existing scales associated with the survey instruments incorporated into baseline and follow-up assessment. For the dietary assessment, no validated tools are available for use in an Australian population so a study-specific tool has been devised. This tool is a 13-item questionnaire addressing dietary quality in this study population and using previously validated questions from the National Nutrition Survey and Cancer Council of Victoria Food Frequency Questionnaire relating to the nutrients of concern for primary prevention of CVD [27,28]. Responses for each item will be scored between zero (least healthy response option) and 10 (healthiest option) - consistent with the developing literature in this area of nutrition research [33]. This scoring allows for an overall diet score to be generated ranging from 0 to 130.

The general quality of dietary intake will be interpreted using the guidance provided in table 1.

**Table 1:** Scoring mechanism used to assess overall quality of dietary intake

Diet score using nutrition tool	Interpretation
104-130	High level of compliance with CVD dietary guidelines (80% or more)

78-103	Medium level of compliance with CVD dietary guidelines (60-79%)

77 or less	Low level of compliance with CVD dietary guidelines (59% or less)

This score allows a good estimate of the level of adherence to key diet-related behaviours. Constituent elements of the 13-item questionnaire also allowed development of sub-scales for these areas of significant influence on cardiovascular health:

• fruit and vegetable intake (soluble fibre and antioxidants)

• saturated fat and total fat intake

• omega-3 fatty acid intake

• fibre intake (insoluble fibre); and

• salt intake.

The sub-scales enable community pharmacists to deliver more tailored dietary advice to participants.

The study protocol was approved by Monash University Human Research Ethics Committee (HREC), University of Tasmania HREC, Flinders University Social and Behavioural Research Ethics Committee and the Australia and New Zealand Clinical Trials Registry (Trial Number ACTRN12609000677202).

## Discussion

The protocol and methods outlined above provide an intervention framework for managing multiple CVD risk factors in community pharmacy or other primary care settings. There is considerable incentive to demonstrate feasibility and effectiveness of such a service in community pharmacy, given the caution with which expanded roles for pharmacists in vascular care have been greeted by some [34]. While there is evidence of pharmacists' ability to conduct cardiovascular risk assessments and deliver basic post-screening counselling with some clinical benefits [35-37], evidence is lacking to demonstrate the full potential of their comprehensive, ongoing management of multiple CVD risk factors. This has led to concerns about fragmentation of care and lack of communication of results with doctors [34].

The HAPA model underpinning the approach to behavioural change was chosen because of its success in lifestyle-oriented diabetes prevention programs addressing similar risk factors for non-diabetic populations, including an implementation study based in Australian primary care [38,39]. Collection of process audit data is important to see how well this approach is adhered to by pharmacists. The emphasis on use of assessment scales for medicines adherence and various health behaviours is designed to promote ease of interpretation of clinical assessments by community pharmacists who are generalists in terms of their practice.

Several aspects of the protocol are context-specific and their adoption by other researchers or health service program managers may require consideration. Key issues include:

• The restriction of eligibility to patients aged 50-74 years is in keeping with national guidelines advocating that screening of overall risk outside the 45-74 years age bracket is not economically viable [9]

• The use of accredited pharmacists has been adopted to integrate with existing pharmacy skills networks and remuneration systems, and to alleviate time pressure for participating community pharmacists; there may be alternative workforce options to produce clinical reports in different settings.

• Patients with established diabetes and CVD will be excluded because there are existing evidence-based models of care already in practice or at implementation stage; and

• Community pharmacy represents a key setting and pharmacists are one of the most accessible healthcare providers in Australia, with expected competencies in both medicines use and health promotion.

### Limitations of study protocol

If the pilot testing indicates the feasibility of effective community pharmacy interventions, it will be important to validate this study with larger numbers and a control group to comprehensively determine effectiveness, cost-effectiveness and generalisability. Similarly, the six month timeframe limits the ability to test the sustainability of patient behaviour changes following the intervention, and self-report - while often the only option - for some health behaviours may be unreliable.

Validated dietary quality tools for cardiovascular health are not available for the Australian population, so we rely on a pilot with only face and content validity developed by one of the authors (SOR). Current Australian guidelines for lipid disorders claim a lack of evidence regarding appropriate cholesterol targets for primary CVD prevention, thus restricting guidance for practitioners. We relied on expert consensus developed from examination of eligibility criteria for subsidised lipid lowering medicines in Australia, previous guidelines, and values used for pathology lab reporting.

## Conclusions

The proposed intervention integrates guideline evidence and validated assessment tools where possible, but several key knowledge gaps remain to preventing an entirely evidence-informed approach. Protocols adopted for other primary CVD prevention studies have not been widely disseminated; hence this paper provides a starting point for future research and implementation programs in a primary care setting.

## Competing interests

The authors declare that they have no competing interests.

## Authors' contributions

KM is lead investigator and wrote the first draft with input from SOR, JD and JG. All authors provided input with subsequent drafts and read and approved the final manuscript.

## Pre-publication history

The pre-publication history for this paper can be accessed here:

http://www.biomedcentral.com/1472-6963/10/264/prepub

## Supplementary Material

Additional file 1 **Clinical reporting form for baseline patient information**. This document is the template used by consultant pharmacists to report baseline findings to pharmacists and doctors. Forms are first provided to the community pharmacist to allow them to add any comments before sending to the doctor. Examples of comments about different aspects of patient health behaviours and treatment are also provided. Click here for file
