# Supplementary material for: Engaging community pharmacists in the primary prevention of cardiovascular disease: protocol for the Pharmacist Assessment of Adherence, Risk and Treatment in Cardiovascular Disease (PAART CVD) pilot study
Source: BMC Health Serv Res. 2010 Sep 7;10:264. doi: 10.1186/1472-6963-10-264 (PMC2941496; doi:10.1186/1472-6963-10-264)
Supplement: Additional file 1 — Clinical reporting form for baseline patient information. This document is the template used by consultant pharmacists to report baseline findings to pharmacists and doctors. Forms are first provided to the community pharmacist to allow them to add any comments before sending to the doctor. Examples of comments about different aspects of patient health behaviours and treatment are also provided. [file 1472-6963-10-264-S1.DOC]

**Additional file 1**

**Title:** Clinical reporting form for baseline patient information

**Description:** This document is the template used by consultant pharmacists to report baseline findings to pharmacists and doctors. Forms are first provided to the community pharmacist to allow them to add any comments before sending to the doctor. Examples of comments about different aspects of patient health behaviours and treatment are also provided.

**Copy of clinical reporting form for general practitioners**

Dear Doctor <Insert name>

As you may be aware your patient, <Insert name and address, DOB> has been enrolled in the Healthy Hearts in Pharmacy Program which is a collaborative project between Monash University, University of Tasmania and the Greater Green Triangle University Department of Rural Health.

This program will target patients from community pharmacies who are being dispensed medications for high blood pressure or high cholesterol, but who have not had a cardiovascular event. Following the initial clinical assessment reported on in this document (including an examination of cholesterol, blood pressure, BMI and lifestyle risk factors), local community pharmacies will implement a support plan offering brief counselling sessions to patients on a monthly basis for six months. They will counsel medication use, medication adherence and lifestyle. We are happy to receive any input you feel is relevant.

Summary of clinical assessment and interpretation

| Parameters | Result |
| --- | --- |
| Smoking status |  |
| Total Cholesterol |  |
| LDL Cholesterol |  |
| HDL Cholesterol |  |
| Triglycerides |  |
| Blood Pressure |  |
| Pulse |  |
| Random Blood Glucose |  |
| Age |  |
| Height |  |
| Weight |  |
| BMI |  |
| Waist |  |
| Family History of CHD |  |
| **Overall cardiovascular risk (% risk of an event over the next five years)** |  |

**Please note: The results from this assessment should be used as a guide only. Suboptimal fasting times can adversely affect lipid results – this patient fasted for (x) hours (optimal 12–16 hours). If you intend to commence and/or modify therapies as a result of this assessment we would advise that the results that we have obtained are repeated by your normal assessment mechanisms.*

Please find attached a current cardiovascular medication list for your patient:

| **Drug prescribed and dose** | **Actual use of medication** |
| --- | --- |
|  |  |
|  |  |

**Medication adherence assessment**

| **Suggestions by accredited pharmacist for implementation and/or follow-up** | **Additional comments by community pharmacist** | **Comments by GP on recommendations** |
| --- | --- | --- |
| Based on the information provided by the patient during the interview and using this patients dispensing history this patient was found to be at risk of suboptimal medication adherence. They expressed reservations about the long term use of these medications. Ongoing reinforcement regarding the benefits of these medications is required, and suboptimal medication adherence may be the reason they have not reached their target levels. |  |  |

**Lifestyle, diet, alcohol, weight management and physical activity based suggestions**

| **Suggestions by accredited pharmacist for implementation** | **Additional comments by community pharmacist** | **Comments by GP on recommendations** |
| --- | --- | --- |
| **Diet**  Mrs X has a diet that has a low level of agreement with foods associated with good cardiovascular health. Through the Healthy Hearts program we will deliver education and motivation to help improve her overall diet. |  |  |
| **Smoking**  As outlined above, Mrs X does want to cease smoking at some stage in the future. We should monitor this and encourage her to give up. |  |  |
| **Physical activity**  Mrs X does not perform adequate physical activity, she undertakes a total of about 30 minutes per week. I will encourage her to gradually increase moderate intensity exercise to 30 minutes five times per week. |  |  |
| **Alcohol**  She does not drink any alcohol |  |  |
| **Weight management**  Her current BMI is 28kg/m^2^. I will encourage her through a program of diet and exercise to reduce her weight and therefore improve her BMI. |  |  |

**Medication based suggestions for possible implementation**

| **Suggestions for follow-up by accredited pharmacist** | **Additional comments by community pharmacist** | **Comments by GP on recommendations** |
| --- | --- | --- |
| **Antiplatelet**  Based on her current level of cardiovascular risk over 5 years, the addition of an antiplatelet agent would appear to be beneficial if she does not have any other contraindications. |  |  |
| **Lipids**  At the time of the interview Mrs X’s lipid profile was just on target for primary prevention. She does not qualify for PBS subsidised lipid lowering, however would benefit from lowering her lipids further through diet and lifestyle and increasing her HDL level. |  |  |

**Ideal targets and suggested patient goals for the six month program:**

1. **Aim to lose weight towards achieving a BMI of 25 and a waist circumference of <80cm.**
2. **Aim to reduce total cholesterol levels to 5.5mmol/L or lower, and TG levels to 1.7mmol/L or lower.**
3. **Aim to increase physical activity to at least 30 minutes on most days.**
4. **Aim to improve diet quality towards healthy eating guidelines**

**In the average person with your results, meeting these targets could help to lower the likelihood of a heart attack or other cardiovascular disease from an estimated (x)% to about (x)% chance over the next five years.**
